# Variability and Predictors of Response to Continuous Theta Burst Stimulation: A TMS-EEG Study

**DOI:** 10.3389/fnins.2018.00400

**Published:** 2018-06-12

**Authors:** Lorenzo Rocchi, Jaime Ibáñez, Alberto Benussi, Ricci Hannah, Vishal Rawji, Elias Casula, John Rothwell

**Affiliations:** ^1^Sobell Department of Motor Neuroscience and Movement Disorders, Institute of Neurology, University College London, London, United Kingdom; ^2^Neurology Unit, Department of Clinical and Experimental Sciences, University of Brescia, Brescia, Italy; ^3^Non-invasive Brain Stimulation Unit, IRCCS Santa Lucia Foundation, Rome, Italy

**Keywords:** transcranial magnetic stimulation, electroencephalography (EEG), TMS-EEG, plasticity, theta-burst stimulation, time-frequency analysis, motor evoked potentials (MEPs)

## Abstract

Continuous theta-burst stimulation (cTBS) is a repetitive transcranial magnetic stimulation paradigm reported to decrease the excitability of the stimulated cortical area and which is thought to reflect a form of inhibitory synaptic plasticity. However, since its introduction, the effect of cTBS has shown a remarkable variability in its effects, which are often quantified by measuring the amplitude of motor evoked potentials (MEPs). Part of this inconsistency in experimental results might be due to an intrinsic variability of TMS effects caused by genetic or neurophysiologic factors. However, it is also possible that MEP only reflect the excitability of a sub-population of output neurons; resting EEG power and measures combining TMS and electroencephalography (TMS-EEG) might represent a more thorough reflection of cortical excitability. The aim of the present study was to verify the robustness of several predictors of cTBS response, such as I wave recruitment and baseline MEP amplitude, and to test cTBS after-effects on multiple neurophysiologic measurements such as MEP, resting EEG power, local mean field power (LMFP), TMS-related spectral perturbation (TRSP), and inter-trial phase clustering (ITPC). As a result, we were not able to confirm either the expected decrease of MEP amplitude after cTBS or the ability of I wave recruitment and MEP amplitude to predict the response to cTBS. Resting EEG power, LMFP, TRSP, and ITPC showed a more consistent trend toward a decrease after cTBS. Overall, our data suggest that the effect of cTBS on corticospinal excitability is variable and difficult to predict with common electrophysiologic markers, while its effect might be clearer when probed with combined TMS and EEG.

## Introduction

Repetitive transcranial magnetic stimulation (rTMS) can produce excitability changes in the stimulated cortical area, which are thought to be linked to synaptic plasticity mechanisms (Ridding and Rothwell, 2007; Ridding and Ziemann, 2010; Censor and Cohen, 2011). Among rTMS protocols, continuous theta-burst stimulation (cTBS) is a fast patterned stimulation able to induce long-term depression-like mechanisms in the cortex (Huang et al., 2005; Suppa et al., 2016). When first introduced, cTBS applied over the primary motor area (M1) was shown to cause a long-lasting depression of motor evoked potentials (MEPs) amplitude, likely due to a decrease in synaptic excitability (Huang et al., 2005). Since then, cTBS has been reported to influence different physiological and behavioral outcomes when applied on a range of cortical areas, both in healthy subject and pathologic conditions (Li Voti et al., 2014; Nardella et al., 2014; Di Biasio et al., 2015; Georgiev et al., 2016; Rocchi et al., 2016). However, a remarkable variability in the effect of cTBS has been found in a large number of experiments, often leading to negative findings (Bologna et al., 2015, 2016; Hannah et al., 2016; Mendez et al., 2017). Various explanations for response variability to cTBS have been proposed; among these there are genetic factors related to receptors and growth factors encoded in the nervous system (Cheeran et al., 2008; Lee et al., 2014), specific interneuronal recruitment by the TMS pulse (Hamada et al., 2013; Hordacre et al., 2017) and baseline size of the probe MEP (Vallence et al., 2015). Whereas subjects’ screening might be the only way to overcome variability due to genetic factors, it is possible to target specific interneuronal cortical populations with TMS or to use an appropriate MEP size by changing coil orientation and stimulation intensity (Hannah et al., 2016). However, only a few studies examined how cTBS effects can be influenced by these variables; thus, further data are needed to investigate the consistency of these predictors.

An interesting question is whether part of the variability in cTBS response is due to the fact that MEP, the most common readout used in the literature, are an incomplete reflection of cortical excitability. Since MEP size is thought to reflect M1 excitability, it is intuitive to think about a parallelism between MEP amplitude and cortical activity, and indeed, MEP amplitude is known to depend on electrical activity of the brain as measured by means of electroencephalography (EEG). In particular, MEP amplitude has been shown to be linked to alpha band power (Sauseng et al., 2009) and beta band power and phase preceding the TMS pulse (Keil et al., 2014; Schulz et al., 2014). However, it is possible that MEP only reflects the excitability of a sub-population of output neurons destined to the spinal cord, failing to reflect all cortical outputs. Moreover, MEPs likely reflect net excitatory and inhibitory inputs to the corticospinal pathway, while paired-pulse paradigms need to be used to effectively probe cortical inhibition separately (Kujirai et al., 1993; Rocchi et al., 2017).

In the last years, many studies investigated the effects of TMS directly from the scalp, in terms of TMS-evoked potentials (TEPs) and oscillations. These indexes have shown a high degree of reproducibility (Lioumis et al., 2009) and might be more representative probes of cortical plasticity since they are not dependent on spinal cord excitability and have been shown to reveal both excitatory and inhibitory cortical activity (Premoli et al., 2014). The effect of cTBS on some of these neurophysiologic variables has already been investigated (Noh et al., 2012; Vernet et al., 2013), although the relationship between variables such as TEP, MEP, and resting EEG is still not clear. Overall, information about the effects of cTBS are still conflicting and a thorough investigation using multiple neurophysiologic outcomes, and the relationships between them, is lacking.

The aim of the present study was to verify whether the effect of cTBS can be predicted by a number of variables related to MEP and already used in past literature. Moreover, we aimed to test cTBS after-effects on multiple measurements (MEP, TEP, resting EEG), assessing the correlation between cTBS effects on MEP, which represent the most common readout for M1 plasticity, and the other neurophysiologic variables.

## Materials and Methods

### Ethics Statement

All procedures were carried out with the adequate understanding and written informed consent of the subjects prior to the experiments. All experimental procedures were conducted in accordance with the Declaration of Helsinki and according to international safety guidelines. Formal approval to conduct the experiments described has been obtained from the human subjects review board of the University College London.

### Subjects

Thirteen healthy subjects (five females, age 27 ± 8), all right handed (Oldfield, 1971) participated in the study. They had no history of neuropsychiatric disorders and were not taking drugs active at the central nervous system level at the time of the experiments. Subjects participated in two experimental sessions using the same cTBS intensity but different with regards to the TMS intensity used, which has been reported to be important for cTBS effects (Vallence et al., 2015). In the first session, they underwent the following baseline (T0) measurements: (A) resting EEG, with eyes open, of approximately 3 min duration; (B) 100 MEPs recorded from the first dorsal interosseous (FDI) muscle and obtained by stimulation of the dominant (left) M1 with posterior-to-anterior (PA) TMS direction, with an intensity suitable to elicit MEP of around 1 mV on average (1 mV-int). EEG was recorded at the same time to obtain TEP; (C) three sets of 20 MEP, recorded from the FDI in PA, anterior-to-posterior (AP) and lateral-to-medial (LM) direction respectively, to characterize the recruitment of different interneuronal populations (see below). After baseline measurements, cTBS was applied over the left M1. After cTBS (T1), measurements A (resting EEG) and B (MEP/TEP) were repeated, approximately within 20 min from cTBS, when the effects of cTBS should be clear (Huang et al., 2005; Rocchi et al., 2016). The second session was similar to the first one, except that in this case a TMS intensity to elicit MEP of around half of the maximum individual amplitude was used (halfmax-int). The order of measurements A (resting EEG) and B (MEP/TEP) was randomized across subjects, both at T0 and T1.

### TMS, Electromyographic Recording, and Analysis

EMG activity was recorded through a pair of Ag/AgCl electrodes placed over the right FDI muscle in a belly-tendon montage. Raw signals were sampled at 5 kHz with a CED 1401 analog-to-digital laboratory interface (Cambridge Electronic Design), amplified and filtered (bandwidth 5 Hz to 2 kHz) with a Digitimer D360 (Digitimer, Ltd.). Data were stored on a laboratory computer for online visual display and additional offline analysis through a dedicated software (Signal, Cambridge Electronic Design). Single-pulse TMS was performed using a Magstim 200 stimulator with a 70 mm figure-of-eight coil (Magstim) that produces stimuli with a monophasic waveform and a pulse width of ∼80 μs.

Repetitive TMS, required for cTBS, was delivered with a biphasic Magstim Rapid2 stimulator according to the standard protocol. Three-pulse bursts at 50 Hz repeated every 200 ms for 40 s were delivered at 80% AMT (Huang et al., 2005). AMT was determined during a 10% maximum voluntary contraction of the right FDI as the lowest magnetic stimulator intensity able to evoke a MEP of at least 200 μV in 5 of 10 consecutive trials. The motor hotspot was defined as the M1 site where TMS evoked the largest MEP in the FDI muscle. Peak-to-peak average amplitudes of the 100 MEPs blocks were calculated and used for subsequent analyses. In the block of 20 MEPs, recorded to characterize I waves recruitment, average latencies were measured and used as the main outcome variables.

### Electroencephalographic Recording and Analysis

Electroencephalography was recorded using a TMS-compatible DC amplifier (ASAlab, ANT Neuro). The amplifier was connected to a PC and the signal was recorded and monitored online through Asalab software (ANT Neuro), and to a 62 channels EEG cap (Waveguard by ANT Neuro). EEG was continuously recorded from 62 TMS-compatible Ag/AgCl pellet electrodes mounted on the cap according to the 10–20 international EEG system, including: Fp1, Fpz, Fp2, F7, F3, Fz, F4, F8, FC5, FC1, FC2, FC6, T7, C3, Cz, C4, T8, CP5, CP1, CP2, CP6, P7, P3, Pz, P4, P8, POz, O1, Oz, O2, AF7, AF3, AF4, AF8, F5, F1, F2, F6, FC3, FCz, FC4, C5, C1, C2, C6, CP3, CPz, CP4, P5, P1, P2, P6, PO5, PO3, PO4, PO6, FT7, FT8, TP7, TP8, PO7, PO8. Recordings were online referenced to linked mastoids and the ground electrode was placed on AFz. In the posterior offline analysis, an average reference was used. Skin impedances were kept below 5 kΩ and the sampling frequency during recording was 2048 Hz. In order to mask the TMS-induced noise and avoid possible auditory ERP, participants wore earplugs continuously playing a white noise mixed with specific time-varying frequencies of the TMS click (Casula et al., 2017b).

Off-line EEG pre-processing was performed with EEGLAB 14.1.1 (Delorme and Makeig, 2004) with the addition of some functions included in the TMS-EEG signal analyzer (TESA) toolbox (Rogasch et al., 2017) and in Fieldtrip open source MATLAB toolbox^1^ (Oostenveld et al., 2011), all running in MATLAB environment (Version 2015b, MathWorks, Inc., Natick, MA, United States).

Resting EEG was band-pass (1–100 Hz) and a band-stop (48–52 Hz) filtered with a fourth order Butterworth filter. An independent component analysis (ICA) algorithm (INFOMAX ICA) was used to visually identify and remove artifacts due to electrode noise, muscle activity and eyeblinks/eye movements. Amplitude values were averaged across the four electrodes around the stimulation site (Fc3, Fc1, C3, C1). Power values in the gamma (30–48 Hz), beta (14–30 Hz), alpha (9–13 Hz), theta (5–8 Hz), and delta (2–4 Hz) frequency bands were calculated by estimating the power spectral density of the signals (Welch’s method, 1 s Hamming windows, no overlapping, 1 Hz resolution). Values were averaged across the four electrodes around the stimulation site (FC3, FC1, C3, C1) and were used for statistical analysis.

Electroencephalography signal recorded during TMS was epoched (-1 to +1 s) and demeaned using a baseline -500 to -10 ms. TMS artifact was removed from -5 to +20 ms around the trigger and interpolated before application of a band-pass (1–100 Hz) and a band-stop (48–52 Hz) fourth order Butterworth filter and again at the end of the pre-processing. The epochs were visually inspected and those with excessively noisy EEG were excluded (less than 5% for each participant). Residual artifacts were identified using an ICA algorithm (INFOMAX ICA) and eliminated after visual inspection, based on time, frequency, scalp distribution, and amplitude criteria (Rogasch et al., 2013, 2014; Casula et al., 2017a).

**FIGURE 1:**
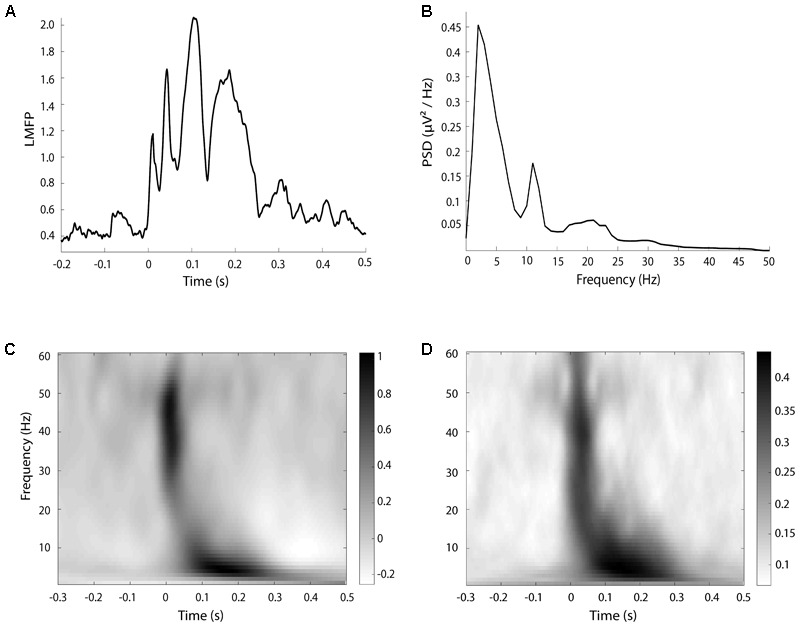
An example of LMFP **(A)**, resting EEG power **(B)**, TRSP **(C)**, and ITPC **(D)**, taken from a single subject in the baseline halfmax-int condition.

Since the aim of the study was to investigate the effects of cTMS at the stimulated site, we calculated several TMS-EEG measures only in a cluster of electrodes surrounding the stimulation site, as for resting EEG. To assess the local cortical activation induced by TMS in the time domain, we computed the local mean field power (LMFP) as the square root of squared TEPs averaged across the four channels of interest, as done in previous paper (Casarotto et al., 2013; Pellicciari et al., 2013; Fecchio et al., 2017). LFMP was measured separately in three time windows (20–70; 70–140; 140–250 ms after the TMS pulse) based on the shape of the LFMP (see section “Results” and **Figure 1**) and the values were used for statistical analysis.

For the time-frequency analysis of TEP, spectral estimations of the EEG epochs were obtained for frequencies between 1 and 60 Hz (1 Hz resolution) and times in the interval from -500 to 500 ms. A sliding window (5 ms steps) linearly increasing its length across frequencies (1 cycle length for 1 Hz up to 7 cycles for 60 Hz) was used to extract the amplitude and power values of all time-frequency bins. These values were estimated using the multitapers method as implemented in fieldtrip’s ft_freqanalysis function. For these estimations, Hanning tapers were used and the amount of spectral smoothing factor was set to 0.1 times the frequency analyzed in each bin. Then, the amount of TMS-related spectral perturbation (TRSP) was computed as follows:

$$TRSP(f\text{​},t)\;\text{=}\;\frac{1}{n}\sum_{k=1}^{n}|F_{k}(f\text{​},t){|}^{2}$$

and inter-trial phase clustering (ITPC) was computed according to the following equation:

$$ITPC(f\text{​},t)\;\text{=}\;\frac{1}{n}\sum_{k=1}^{n}\frac{F_{k}(f\text{​},t)}{\left|F_{k}(f\text{​},t)\right|}$$

where, for *n* trials, the spectral power/amplitude estimates *P* and *F* were computed at trial *k*, at frequency *f* and time *t* (Delorme and Makeig, 2004). TRSP was evaluated locally by averaging the values obtained by the electrodes surrounding the stimulation site (FC3, FC1, C3, C1). For the statistical analysis, TSRP values were averaged from 20 to 70 ms for gamma (31–48 Hz) and beta (14–30 Hz) frequency bands, and from 70 to 300 ms for alpha (8–13 Hz), theta (5–8 Hz), and delta (2–4 Hz) bands, based on time distribution of TRSP and ITPC. The choice of the time windows was based on the timing of frequency response after the TMS pulse (see section “Results” and **Figure 1**).

### Characterizing the Recruitment of Different Interneuron Populations

The corticospinal pathway responds to supra-threshold TMS with a series of descending volleys known as indirect waves (I-waves) which are thought to result from *trans-*synaptic activation of the corticospinal cells. The first descending I-waves have a lower threshold for activation and preferentially respond to currents directed across the central sulcus in the PA direction, whilst later I-waves have a higher threshold and are preferentially recruited by AP-directed currents (Di Lazzaro et al., 2004). They are therefore thought to reflect activity in different excitatory interneuronal circuits. Previous studies have measured the onset latency of MEPs recorded in a hand muscle as a surrogate for I-waves (Day et al., 1989; Hamada et al., 2013; Hannah and Rothwell, 2017), which can only be recorded invasively in the spinal epidural space. Here, we measured the onset latency of MEPs to AP stimuli as a marker of the preferential recruitment of early or late I-waves. These were compared to the latency of MEPs evoked by direct wave recruitment though latero-medial (LM) currents. The rationale is that individuals in whom early I-waves are preferentially recruited will show little difference in the onset latency of AP and LM evoked MEPs, whereas individuals in whom late I-waves are preferentially recruited will show a larger difference (Hamada et al., 2013).

### Statistical Analysis

Baseline TMS measures common to the two experimental sessions (AMT obtained with the biphasic stimulator and AMT measured with the monophasic stimulator, the latter both in PA and AP coil direction) were compared by means of paired *t*-tests. Two paired *t*-tests were used to investigate the effects of cTBS on MEP amplitude in 1 mV-int and halfmax-int conditions. Several two-way repeated measures ANOVAs were performed to investigate possible effects of cTBS in the two experimental sessions (implying a different TMS intensity for all examined variables except resting EEG power) separately. The first factor was “time” (T0, T1) and the second varied according to the variable investigated. Specifically, it was “time of interest” (ToI) (20–70, 70–140, 140–250 ms) for LMFP, while it was “frequency of interest” (FoI) (for delta = 2–4, theta = 5–8, alpha = 9–13, beta = 14–30, gamma = 30–48 Hz) for TRSP, ITPC, and power in resting EEG. Where the results of *t*-tests/ANOVAs suggested no statistically significant effects of cTBS, an equivalent Bayesian test (*t*-test or ANOVA) was performed to provide more evidence about whether the null hypothesis was true. A Pearson’s correlation coefficient was used to check (1) whether latency of AP MEP, as well as the latency difference between AP and LM MEP, was correlated to response to cTBS, defined as the ratio between MEP amplitude at T1 on T0, as reported previously (Hamada et al., 2013; Hordacre et al., 2017), (2) cTBS induced changes in MEP correlated with changes in LMFP, TRSP, and ITPC within the same session, and (3) cTBS induced correlated changes in the same outcome measures (MEP, resting EEG power, LMFP, TRSP, ITPC) across the two different sessions. Before undergoing ANOVA procedures, normal distribution of data was assessed by means of Shapiro–Wilk’s test. All *p*-values < 0.05 were considered significant. Greenhouse–Geisser correction was used when necessary to correct for non-sphericity (i.e., Mauchly’s test < 0.05). To correct for multiple comparisons, Bonferroni’s correction was used for all *post hoc* analyses following the ANOVA and in the correlation tests. Statistical analyses were performed with IBM SPSS v24 or JASP v0.8.6.

## Results

Overall, the test sessions were well-tolerated and no participants reported any side effects. Baseline TMS parameters are summarized in **Table 1**. Visual examples of LMFP, TRSP, ITPC, and power spectrum of resting EEG are given in **Figure 1**. AMT measured with both biphasic and monophasic stimulators, as well as in both PA and AP coil directions, were not different in the two sessions (all *p*-values > 0.05) (**Table 1**). When only the factors “ToI” or “FoI” were significant, *post hoc* analyses were not done because differences in the explored variables regardless of the effect of cTBS, were not considered relevant for the study.

**Table 1:** Summary of main baseline neurophysiologic values, expressed as average ± standard deviation.

Ses	SI	MEP amp	AMT_bi_	AMT_mono_ PA	AMT_mono_ AP	AP lat	PA lat	LM lat
1 mV	61.39 ± 7.88	1.32 ± 0.66	59.10 ± 6.96	40.08 ± 5.16	52.38 ± 10.5	24.06 ± 2.64	22.06 ± 1.55	20.75 ± 1.51
HM	68.0 ± 11.34	2.59 ± 1.51	57.37 ± 8.71	38.92 ± 5.30	52.84 ± 11.64	24.13 ± 2.39	21.99 ± 1.58	20.99 ± 1.52

*Thresholds (*AMT_*bi*_, AMT_*mono*_* PA, *AMT_*mono*_* AP) are expressed in percentage of the maximal stimulator output. MEP amplitude is expressed in mV. AP, PA, and LM latencies are measured in ms. *AMT_*bi*_*, active motor threshold obtained with biphasic TMS stimulator; *AMT_*mono*_*, active motor threshold obtained with monophasic TMS stimulator; *HM*, halfmax-int (see text); *Ses*, session; *SI*, stimulation intensity.*

**FIGURE 2:**
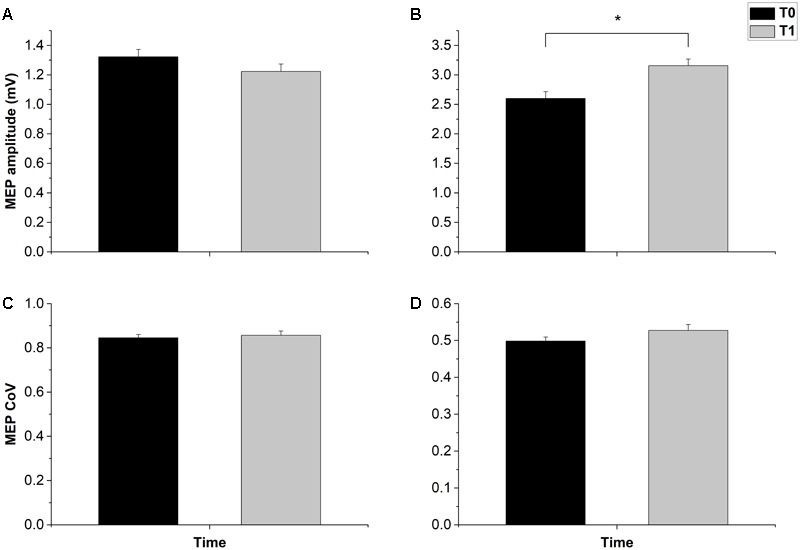
Effects of cTBS on MEP amplitude and MEP CoV. cTBS had no effect on both MEP amplitude and CoV in the 1 mv-int condition **(A,C)**. In the halfmax-int session cTBS induced a significant amplitude increase of MEP **(B)**, while MEP CoV was not significantly changed **(D)**. Error bars represents standard error. Asterisks indicate statistical significance (*p* < 0.05).

Overall, the MEP results were inconsistent with the prototypical responses to cTBS, being either unchanged or facilitated, instead of inhibited (Huang et al., 2005). The *t*-test on the MEP amplitude in the 1-mV session showed a null group effect (*t*_12_ = 0.995, *p* = 0.339). The result was confirmed by a Bayesian paired-*t* test, which showed a Bf of 0.286, thus strongly supporting the null hypothesis. By contrast, in the halfmax-int condition MEP amplitude was significantly increased (*t*_12_ = 2.838, *p* = 0.01) (**Figure 2**). Main effects and interactions of the ANOVAs are summarized in **Table 2**.

**Table 2:** Summary of neurophysiologic outcomes in both sessions and *F*- and *p*-values of the related ANOVAs.

Variable	Session	Time			ToI or FoI			Interaction		
		*F*	df, e	*p*	*F*	df, e	*p*	*F*	df, e	*p*
LMFP	1 mV	1.69	1,12	0.22	1.46	2,24	0.25	0.67	2,24	0.52
	HM	3.97	1,12	0.069	0.861	2,24	0.435	0.269	2,24	0.766
TRSP	1 mV	0.47	1,12	0.503	6.349	4,48	<0.01	2.354	4,48	0.067
	HM	6.212	1,12	0.028	2.827	4,48	0.035	7.613	4,48	<0.001
ITPC	1 mV	3.372	1,12	0.091	2.503	4,48	0.055	1.322	4,48	0.275
	HM	1.410	1,12	0.258	3,482	4,48	0.014	5.502	4,48	0.001
REP	1 mV	0.444	1,12	0.518	15.2	4,48	<0.001	3.426	4,48	0.047
	HM	2.383	1,12	0.149	19.795	4,48	<0.001	3.718	4,48	0.023

*Significant *p*-values (<0.05) are underlined.*

**FIGURE 3:**
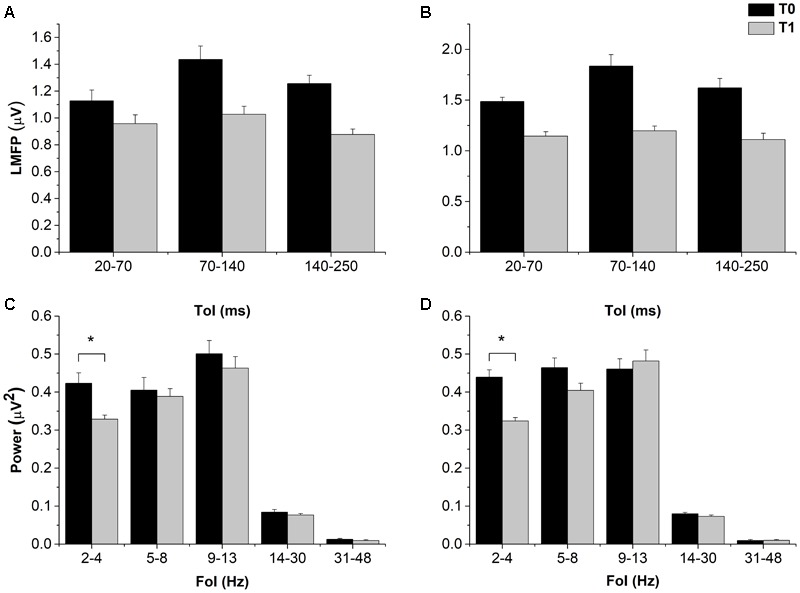
Effects of cTBS on LMFP and power of resting EEG. Despite a trend toward a decrease in both 1 mV-int **(A)** and halfmax-int **(B)** sessions, cTBS did not induced statistically significant changes in LMFP in all the explored ToI. By contrast delta band (2–4 Hz) power was decreased after cTBS in both sessions **(C,D)**. MEP amplitude and MEP CoV Error bars represents standard error. Asterisks indicate statistical significance (*p* < 0.05).

By contrast, resting EEG power and TMS-EEG measures globally showed a rather consistent inhibition, with variable outcomes depending on the ToI, FoI, and TMS intensity used. LMFP showed a clear trend toward a decrease after cTBS in both the 1 mV-int and halfmax-int condition, and in all the time windows explored, although the main effects and the interactions between them were not significant (**Figure 3**). The trend was confirmed by the Bayesian ANOVAs, which showed a Bf of 2.279 and 2.798 for factor “time” in the 1 mV-int and halfmax sessions respectively. The implication is that there is slight evidence for adding “time” to the null model to explain our results.

**FIGURE 4:**
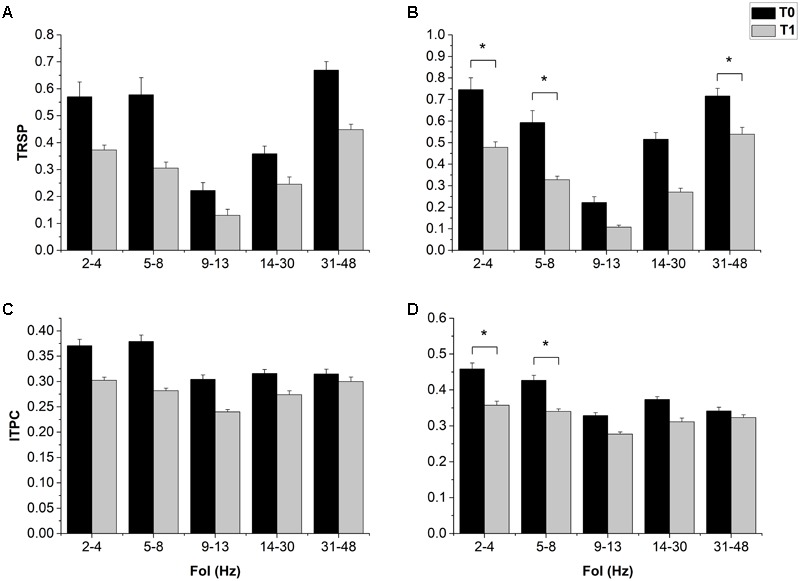
Effects of cTBS on LTRSP and ITPC. cTBS only induced a trend toward a decrease in all FoI in both TRSP **(A)** and ITPC **(C)**. In the halfmax-int session, cTBS induced a decrease in delta (2–4 Hz) and theta (5–8 Hz) in both TRSP **(B)** and ITPC **(D)**; only in TRSP there was also a significant decrease in the gamma band (42–48 Hz). Error bars represents standard error. Asterisks indicate statistical significance (*p* < 0.05).

The ANOVA on resting EEG power in the 1 mV-int condition showed a significant effect of “FoI” and a significant “Time × FoI” interaction; *post hoc* analyses showed a significant decrease in power in the delta band after cTBS (*p* = 0.023), and this was confirmed in the halfmax-int session (*p* = 0.010) (**Figure 3**). The ANOVA on TRSP in the 1 mV-int condition showed a trend toward a decrease after cTBS, although this was not statistically significant. By contrast, the ANOVA on TRSP in the halfmax-int condition showed a significant main effect of “time,” “Foi,” and a significant “time × FoI” interaction. *Post hoc* analyses showed a significant decrease in TRSP in the delta, theta, and gamma band (*p* = 0.022, *p* < 0.001, and *p* < 0.01, respectively) (**Figure 4**). ITPC in the 1 mV-int condition showed a trend toward a decrease after cTBS, although there were no significant main effects or interactions. However, the ANOVA on halfmax-int condition showed significant main effects of “time,” “FoI” and a significant interaction between them. *Post hoc* analyses showed that ITPC in the delta and theta bands was significantly lower after cTBS (*p* = 0.019 and *p* = 0.013, respectively) (**Figure 4**).

Finally, Pearson’s correlation test disclosed no significant correlation (all *p*-values > 0.05). Thus, no correlation was observed between response to cTBS and the latency of AP MEP or the latency difference between AP and LM MEP. Additionally, no correlation was observed between MEP and EEG/TMS-EEG measures in the same session, and the effect of cTBS on all tested outcomes (MEP, resting EEG power, LMFP, TRSP, ITPC) was not consistent across the two sessions.

## Discussion

In this paper, we were not able to replicate previous findings about cTBS effects on MEP amplitude and on predictors of response to cTBS. Specifically, cTBS did not decrease MEP amplitude when they were about 1 mV, and it unexpectedly increased their amplitude when it was around half of its maximum at baseline. I-wave recruitment was not correlated to cTBS response. However, the effect of cTBS seemed to be clearer on purely cortical electrophysiological markers, i.e., cTBS globally reduced LMFP and decreased TRSP, resting EEG power and ITPC, especially in low-frequency bands. The effects ranged from trends to statistical significance, especially when a higher TMS intensity was used for TRSP and ITPC, as outlined in the results. Overall, our data suggest that the effects of cTBS on corticospinal excitability are variable and difficult to predict with common electrophysiologic markers, while its effect might be clearer when probed with combined TMS and EEG.

### Motor Evoked Potentials and Predictors to cTBS Response

In our sample of subjects, and the 1 mV-int condition, cTBS was not able to decrease MEP amplitude as was described in the original report (Huang et al., 2005). However, since then, there have been many reports about null effects of cTBS. In particular, Hamada et al. (2013) found no group effect of cTBS on a sample of more than 50 subjects; in this view, our result is not surprising. However, it is more difficult to explain why we found a significant increase in MEP amplitude in the halfmax-int condition, especially considering that, according to the report by Vallence et al. (2015), the inhibitory effect of cTBS should be clearer when MEP have a baseline amplitude similar to that used here. We used a large number of MEP in our investigation, thus reducing the possibility of MEP variability due to changing brain states. Considering that a high variability in the effects of cTBS on MEP was described in a large sample of subjects (Hamada et al., 2013), it is possible that our sample mostly included “opposite responders” to cTBS, possibly due to genetic factors (Jannati et al., 2017). Additionally, our marker of late I-wave recruitment (AP latency and AP-LM latency difference) did not correlate with cTBS after effects, in contrast with what was previously reported (Hamada et al., 2013). Again, as for cTBS effects, our results are difficult to explain, except in the light of the known variability in cTBS, which might at least partly be independent from the studied predictors.

### Spontaneous and TMS-Evoked EEG Activity

One possibility to explain our null findings might be that the readout itself, the MEP, is not optimal for detecting cortical excitability changes induced by rTMS. Indeed, it is known that MEP show a large, and relatively unexplained, intertrial variability (Kiers et al., 1993; Magistris et al., 1998) and that their amplitude is partly dependent on spinal motoneuron excitability (Ellaway et al., 1998; Funase et al., 1999). By contrast with the MEP, the EEG-derived measures only depend on activity in cortical circuitry. LMFP represents the standard deviation of the EEG voltage difference induced by an event (the TMS pulse, in this case), measured in a cluster of electrodes of interest, and as such, it quantifies the amount of activity at each time point in the field considered, resulting in a reference-independent descriptor of the potential field (Lehmann and Skrandies, 1980; Skrandies, 1990). LMFP in both sessions showed a clear trend toward a decrease in all the time windows explored, consistent with the “expected” outcomes of cTBS. However, the effect was not statistically significant due to a high variability across subjects. By contrast, statistics on TRSP showed clearer results, especially in the halfmax-int session, consisting in a significant decrease in TRSP in the delta, theta, and gamma frequency bands. TRSP (Pfurtscheller and Aranibar, 1979; Delorme and Makeig, 2004) represents event-related changes in spectral power over time in a broad frequency range. As such, it takes into account the phase-locked and non-phase locked EEG perturbations induced by TMS. This is different from time-domain measures, where non-phase locked activity cancels out. This might explain why the effect of cTBS on TRSP, despite being similar to that on LMFP, was clearer. This result was coherent with the decrease in delta band power we found on resting EEG; notably, this was reproduced in both sessions with the same stimulation and recording conditions. In our setting, ITPC was used to assess synchronization of activity at a particular latency and frequency after the TMS pulse (Tallon-Baudry et al., 1996; Delorme and Makeig, 2004). We found that ITPC was reduced in the delta and theta frequency range and, similar to TRSP, although the effect was present in both sessions, it reached statistical significance only in the halfmax-int condition.

Overall, we found that cTBS decreased power and synchronization of spontaneous and evoked EEG activity, confirmed by different measures in two experimental sessions. One mechanism by which cTBS might have influenced our measures is by increasing neural “noise” in the stimulated area (Ruzzoli et al., 2010; Teo et al., 2011; Rocchi et al., 2016). This increase in noise might have caused an increased jitter in spontaneous or induced activity of neural ensembles close to the stimulation site; this reduced degree of synchronicity might justify the lower REP, TRSP, and ITPC (Musall et al., 2014). However, the results were not completely homogeneous across all bands of activity. Power decreases in resting EEG occurred only in the delta band, whereas the decrease in TRSP and ITPC also occurred in the theta band. The reason for this is not clear, but it might be due to the characteristics of the probe used. In fact, TRSP and ITPC test cortical perturbation induced by a repetitive and consistent stimulation, thus increasing signal to noise ratio by a large, synchronized input, while resting EEG contains a variable and heterogeneous mix of information and noise which might be less suited to probe plasticity effects (Yarom and Hounsgaard, 2011). Also, the effect of cTBS on the gamma band shows a different effect when comparing TRSP and ITPC, i.e., TRSP decreased whereas ITPC did not change significantly. The reason might be that gamma TRSP takes place mainly in the first 100 ms from the event, and thus it might be more strictly phase locked to the TMS pulse. Alternatively, this discrepancy might be ascribed to different baseline levels, i.e., TRSP and ITPC showed modulation after cTBS only in the frequency bands where they reached higher levels (**Figures 3, 4**); in this view, unaffected frequency bands might not have been changed due to a floor effect. Lastly, significant effects with regards to TRSP and ITPC were observed only when using a TMS intensity able to induce an MEP of around half of its maximum. There are no studies systematically comparing the effects on cTBS on TRSP and ITPC obtained with different TMS intensities; the most parsimonious explanation is that the intensity we used in the first session was not sufficient for clear effects to be observed.

As it was for MEP, our results TMS-evoked EEG activity are partly at odds with the ones present in past literature. Vernet et al. (2013) found that, after cTBS, TRSP was lower in theta and alpha bands, but it was higher in the high beta range. Noh et al. (2012), by contrast, showed an increased in theta and beta TRSP. The main technical factor limiting a possible comparison is that in our study we assessed TRSP only in time windows relevant to each frequency band (see section “Materials and Methods” and **Figure 1**), while in the mentioned studies TRSP was averaged across the whole epoch segment after TMS (1 s), thus introducing a considerable amount of noise into the analysis. The present findings on spontaneous EEG activity are somewhat at odds with previous reports as well. A decrease in beta power and an increase in theta power after cTBS was reported by Vernet et al. (2013); notably, subjects had their EEG recorded with their eyes closed, which might possibly have led to results different than ours by altering the baseline power of different EEG bands. McAllister et al. (2011) found no effects of cTBS on EEG power, suggesting that EEG itself might not be the best tool to investigate the effect of plasticity-inducing TMS protocols. However the authors recorded EEG with a single central bipolar channel; given that activity in low frequency bands, such as delta, tends to be diffuse, it is possible that without a large number of electrodes and an average reference a possible difference in power induced by cTBS might have been overlooked.

### Correlation Between MEP and TMS-EEG Measures

We found no correlation between MEP and any of our TMS-EEG measures (LMFP, TRSP, ITPC) or resting EEG power in either experimental sessions. Again, data in past literature are conflicting. For example, Paus et al. (2001) showed a correlation with amplitude of N100 TEP components and MEP amplitude, while Mäki and Ilmoniemi (2010) found the same correlation with N15-P30 TEP components; however other authors could not replicate this result (Bender et al., 2005; Bonato et al., 2006; Van Der Werf and Paus, 2006). Part of this discrepancy might be due to difficulties in understanding the sources of these TEP components, which have been reported to vary across areas others than M1 (Komssi et al., 2002; Esser et al., 2006; Litvak et al., 2007); thus, they might not be necessarily informative about the dynamics underlying MEP generation, which are thought to take place within M1 during the first few ms after the TMS pulse (Di Lazzaro et al., 2004). However, it should be noted that the TMS artifact interpolation used here (20 ms) might have shadowed at least the N15 component, thus limiting the possibility of finding a correlation in this time range.

Overall, the present data show that several published neurophysiologic predictors to cTBS response are not robust when small sample of subjects are studied, and that cTBS effects as measured by MEP can be unexpected. By contrast, resting EEG power, TRSP and ITPC seem to show effects which are more consistent and more in line with the expected effects of cTBS, and thus should probably be investigated more thoroughly in the context of rTMS.

## Author Contributions

LR: conception and design of the work; acquisition, analysis, and interpretation of data; and drafting the work. JI, AB, RH, and VR: conception and design of the work; analysis and interpretation of data; and drafting the work. EC: conception and design of the work; analysis and interpretation of data; and revision of the work. JR: conception of the work; interpretation of data; and revision of the work.

## Conflict of Interest Statement

The authors declare that the research was conducted in the absence of any commercial or financial relationships that could be construed as a potential conflict of interest. The handling Editor declared a past co-authorship with one of the authors EC.

## Footnotes

1 www.ru.nl/fcdonders/fieldtrip/
